# Identifying Early Changes in Myocardial Microstructure in Hypertensive Heart Disease

**DOI:** 10.1371/journal.pone.0097424

**Published:** 2014-05-15

**Authors:** Pranoti Hiremath, Michael Bauer, Aaron D. Aguirre, Hui-Wen Cheng, Kazumasa Unno, Ravi B. Patel, Bethany W. Harvey, Wei-Ting Chang, John D. Groarke, Ronglih Liao, Susan Cheng

**Affiliations:** 1 Harvard Medical School, Boston, Massachusetts, United States of America; 2 The Department of Medicine, Brigham and Women's Hospital, Boston, Massachusetts, United States of America; 3 The Cardiovascular Division, Brigham and Women's Hospital, Harvard Medical School, Boston, Massachusetts, United States of America; Tokai University, Japan

## Abstract

The transition from healthy myocardium to hypertensive heart disease is characterized by a series of poorly understood changes in myocardial tissue microstructure. Incremental alterations in the orientation and integrity of myocardial fibers can be assessed using advanced ultrasonic image analysis. We used a modified algorithm to investigate left ventricular myocardial microstructure based on analysis of the reflection intensity at the myocardial-pericardial interface on B-mode echocardiographic images. We evaluated the extent to which the novel algorithm can differentiate between normal myocardium and hypertensive heart disease in humans as well as in a mouse model of afterload resistance. The algorithm significantly differentiated between individuals with uncomplicated essential hypertension (N = 30) and healthy controls (N = 28), even after adjusting for age and sex (P = 0.025). There was a trend in higher relative wall thickness in hypertensive individuals compared to controls (P = 0.08), but no difference between groups in left ventricular mass (P = 0.98) or total wall thickness (P = 0.37). In mice, algorithm measurements (P = 0.026) compared with left ventricular mass (P = 0.053) more clearly differentiated between animal groups that underwent fixed aortic banding, temporary aortic banding, or sham procedure, on echocardiography at 7 weeks after surgery. Based on sonographic signal intensity analysis, a novel imaging algorithm provides an accessible, non-invasive measure that appears to differentiate normal left ventricular microstructure from myocardium exposed to chronic afterload stress. The algorithm may represent a particularly sensitive measure of the myocardial changes that occur early in the course of disease progression.

## Introduction

Hypertensive heart disease is characterized by remodeling of the myocardium in response to chronically elevated blood pressure and wall stress.[1] Histologic studies suggest that hypertensive changes of the myocardium typically involve an increase in cardiomyocyte size in addition to deposition of extracellular matrix and fibrosis of the interstitium.[2] Specialized imaging techniques offer the ability to non-invasively investigate these microstructural changes in both experimental models and clinical studies. Whereas conventional echocardiographic measures capture features of gross (i.e. ‘macroscopic’) structural change, such as increased left ventricular (LV) wall thickness and mass, advanced ultrasonic imaging methods may capture alterations in the microstructure, reflecting myocardial tissue and cellular morphology, and offer additional complementary information regarding the cardiac response to chronic afterload stress.

Ultrasonic indices may be able to distinguish pathological tissue properties including myocardial disarray, size heterogeneity, increased myocyte density, and interstitial fibrosis.[3], [4] Investigators have hypothesized that these properties alter tissue impedances, thereby changing sonographic signal reflections and yielding signal intensity distributions that are unique to the scattering properties of the imaged LV wall. Thus, prior studies have used sonographic image analysis, including integrated backscatter techniques, to characterize myocardial tissue alterations in a variety of clinical settings, including hypertension,[5] early myocardial infarction,[6] chronic coronary artery disease,[7] and hypothyroidism.[8], [9] However, the use of previously established techniques has been limited by low sensitivity and specificity, particularly in the setting of poorer quality images.[10] Integrated backscatter techniques, for example, depends predominantly on mean signal values and has demonstrated variability due to random noise,[11] susceptibility to time delays arising from the application of algorithms,[12], [13] and limited correlation with the extent of myocardial fibrosis present.[10] Therefore, we assessed the ability of a modified ultrasound-based image analysis algorithm to quantify LV wall microstructural alterations using distributions of sonographic signal intensity values. We evaluated this algorithm in humans with variable exposure to elevated blood pressure, as well as in a mouse model of chronic afterload stress.

## Materials and Methods

### Image Analyses

We developed a computational method of analysis using the ImageJ software platform v1.46 (NIH, Bethesda, MD) to perform measurements of signal intensity distributions within 2D ultrasound images. We created an ImageJ macro to analyze a user-selected region-of-interest within an 8-bit DICOM echocardiographic image or an 8-bit .jpg image, with individual pixels ranging from 0 to 255 intensity.[14] The user protocol has been standardized for end-diastolic frames of B-mode long-axis left ventricular human and murine echocardiograms, including anatomical markers for placement of myocardial and adjacent pericardial regions of interest (see **Supporting Information File S1**). The algorithm functions to hierarchically order pixels within the region of interest, and then provide characteristics of the signal intensity distribution. The general region of interest was defined as the inferolateral mid-to-basal myocardial segment of the LV in the parasternal long-axis view (**Figure S1** in **File S1**); the specific region of interest was defined as the pericardium adjacent and parallel to the mid-to-basal section of the inferolateral segment of the myocardium. The Signal Intensity Coefficient (SIC) was calculated as (1-p/256), where p is the 25^th^ percentile of pericardial signal intensity distribution; the SIC demonstrates limited variation with change in image acquisition gain (**Figure S2** in **File S1**). Intra-class correlation coefficients for inter-reader and intra-reader reproducibility are 0.89 and 0.90, respectively.

In addition to the advanced image analyses described above, we also assessed conventional echocardiographic measures of structure and function including: LV wall thickness (LVWT) calculated as interventricular septum width plus posterior wall width, LV end-diastolic diameter (LVDD), LV end-systolic diameter (LVSD), LV mass, LV fractional shortening (FS), LV ejection fraction (calculated from the aforementioned linear measurements using the Teiccholz method),[15] peak E prime velocity (lateral annulus), peak transmitral E velocity to A velocity ratio, and peak transmitral E velocity to peak E prime velocity ratio. The relative wall thickness (RWT) was calculated as LVWT divided by LVDD.

To capture information regarding both the microstructural and gross (i.e. ‘macroscopic’) morphological changes that can occur early in the progression of hypertensive heart disease, we also calculated an aggregate measure referred to as the myocardial structural index (MSI) and defined the MSI as equal to SIC/0.13+RWT/0.05 (i.e. the sum of the standardized value of SIC and the standardized value of RWT).

### Clinical Study Sample

From a database of routine echocardiographic studies performed on patients referred to our institution's clinical laboratory, we selected echocardiograms from normotensive healthy controls (N = 28) and individuals with hypertension (N = 30) without diabetes or prevalent cardiovascular disease (i.e. free of coronary disease, heart failure, or prior cerebrovascular event). All images underwent review, while blinded to clinical data, for image quality and appropriateness for image analysis (based on visualization of the endocardial border in relation to the LV cavity and adjacent structures). In addition to conventional echocardiographic measures,[16] the advanced image analysis algorithm (including calculation of the SIC) was performed on all images in a blinded fashion. Data on clinical characteristics were extracted from electronic medical record, including blood pressure measurement performed on the day of or closest to the date of echocardiographic image acquisition (13±14, maximum 45 days). The following blood pressure measurements were recorded: systolic blood pressure (SBP), diastolic blood pressure (DBP), and mean arterial pressure (MAP) calculated as DBP plus (SBP-DBP)/3. All patient records and information were anonymized and de-identified prior to analyses. All clinical research protocols and procedures were approved by the institutional review board of the Brigham and Women's Hospital.

### Experimental Study

We studied a cohort of 12 adult mice (C57/BL6, Jackson Laboratory) that were housed in a climate controlled facility with 12-hour alternating light cycles and access to food and water *ad libitum*. All animals were studied over a period of 7 weeks, after being randomized into one of the following 3 groups: vehicle control with sham operation at baseline (N = 3), ascending aortic constriction applied surgically at baseline (N = 4), and ascending aortic constriction applied surgically at baseline with subsequent removal of the aortic band at 3 weeks (N = 3). Details regarding the experimental protocol, including the surgical aortic banding procedures, have been described previously.[17] At 7 weeks, all mice underwent echocardiographic image acquisition using a 28 MHz transducer with digital image capture (Vevo2100 Visualsonics, Toronto, ON) according to a standardized protocol.[18] All images underwent quality review (based on the criteria described above) and then advanced image analysis, including calculation of the SIC, in a blinded fashion. At 7 weeks, following echocardiography, mice were euthanized for pathological assessment of myocardial fibrosis. Histologic quantification of myocardial fibrosis was performed using a previously described automated image-analysis method (ImageJ, v1.46, Bethesda, MD).[19] All animal procedures were approved by the Harvard Medical Area standing Institutional Animal Care and Use Committee.

### Statistical Analyses

For analyses in humans, we compared clinical and echocardiographic characteristics of normotensive versus hypertensive individuals using the two-sided Student's T-test for continuous variables and the chi-square test for categorical variables. We then used regression analyses to examine the association of both conventional and advanced echocardiographic parameters (including both SIC and MSI) with hypertension status (present versus absent). In addition to unadjusted models, we performed regression analyses adjusting for age and sex. We also examined the association of conventional and advanced echocardiographic measures with increasing tertiles of each BP measure (SBP, DBP, and MAP). Additionally, in the total sample of individuals studied, we used regression analyses to examine the association of variation in SBP, DBP, and MAP with both conventional and advanced echocardiographic measures of LV structure (including both the SIC and MSI).

For the experimental study, we assessed differences in LV mass, RWT, SIC, and MSI at 7 weeks across all animal groups using the Kruskal-Wallis test, and between pairs of groups using the two-sided Student's t-test.

All analyses were performed using Stata SE (v12.1, StataCorp, College Station, TX), and a 2-sided P value<0.05 was considered statistically significant.

## Results

### Clinical Study Results

Clinical and echocardiographic characteristics for individuals in the healthy control group (N = 28) compared with individuals in the hypertensive group (N = 30) are shown in **Table 1**. There was no significant difference in age or sex when individuals in the healthy control group were compared to those in the hypertensive group (P = 0.24). As expected, all BP measures were significantly higher in the hypertensive group. With respect to echocardiographic traits, there was no significant difference between groups in conventional measures of LV structure and systolic function; the conventional measures of diastolic function, E prime and E/e' ratio, were worse in hypertensives than controls, as expected (**Table 1**). The novel measure of myocardial microstructure, SIC, was also significantly higher in hypertensives compared with normotensives (P = 0.029) as was the MSI (P = 0.008). Accordingly, in regression analyses (**Table 2**), conventional echocardiographic parameters were not significantly associated with hypertensive status in models with and without adjustment for age and sex, with the exception of E prime and E/e' ratio. Notably, higher values of SIC (P = 0.029) and MSI (P = 0.008) were also associated with hypertension in unadjusted models; these associations remained significant in models adjusting for age and sex.

**Table 1:** Characteristics of the Clinical Study Sample.

Characteristics	Healthy Controls (N = 28)	Hypertensives (N = 30)	P value
***Clinical***			
Age	52±9	54±3	0.24
Women, %	56	47	0.34
Systolic blood pressure, mmHg	121±9	142±19	<0.001
Diastolic blood pressure, mmHg	72±9	85±12	<0.001
Mean arterial pressure, mmHg	112±10	132±16	<0.001
***Echocardiographic***			
LV wall thickness, cm	1.8±0.2	1.9±0.3	0.33
LV end-diastolic diameter, cm	4.0±0.5	4.0±0.5	0.87
LV mass, g	146±56	149±44	0.84
Relative wall thickness	0.45±0.01	0.47±0.07	0.079
Fractional shortening	0.28±0.08	0.27±0.15	0.86
Ejection fraction, %	62±4	63±3	0.35
E prime, cm/s	13.2±3.1	10.1±2.8	0.0002
E/a ratio	1.2±4.2	1.1±4.0	0.36
E/e' ratio	6.2±1.6	7.5±2.1	0.014
Signal intensity coefficient	0.23±0.11	0.31±0.15	0.029
Myocardial structural index	10.7±0.89	11.7±1.79	0.008

Values are shown as means±standard deviations or percent frequencies.

**Table 2:** Associations of Echocardiographic Parameters with Hypertension Status.

	Hypertension Status	
	Est. Coeff (SE)*	P value
***Unadjusted***		
Left ventricular wall thickness, cm	0.066 (0.067)	0.33
Left ventricular end-diastolic diameter, cm	−0.011 (0.067)	0.87
Left ventricular mass, g	0.013 (0.067)	0.84
Relative wall thickness	0.117 (0.066)	0.079
E prime, cm/s	−0.236 (0.059)	0.0002
E/a ratio	−0.062 (0.067)	0.36
E/e' ratio	0.161 (0.064)	0.014
Signal intensity coefficient	0.144 (0.065)	0.029
Myocardial structural index	0.175 (0.063)	0.008
***Adjusted for age and sex***		
Left ventricular wall thickness, cm	0.071 (0.078)	0.37
Left ventricular end-diastolic diameter, cm	−0.035 (0.079)	0.66
Left ventricular mass, g	−0.002 (0.081)	0.98
Relative wall thickness	0.118 (0.065)	0.076
E prime, cm/s	−0.272 (0.070)	0.0003
E/a ratio	−0.050 (0.068)	0.47
E/e' ratio	0.153 (0.071)	0.036
Signal intensity coefficient	0.153 (0.066)	0.025
Myocardial structural index	0.179 (0.063)	0.007

*Estimated regression coefficients represent the change in blood pressure measure per 1-SD change in the echocardiographic parameter.

**Figure 1:**
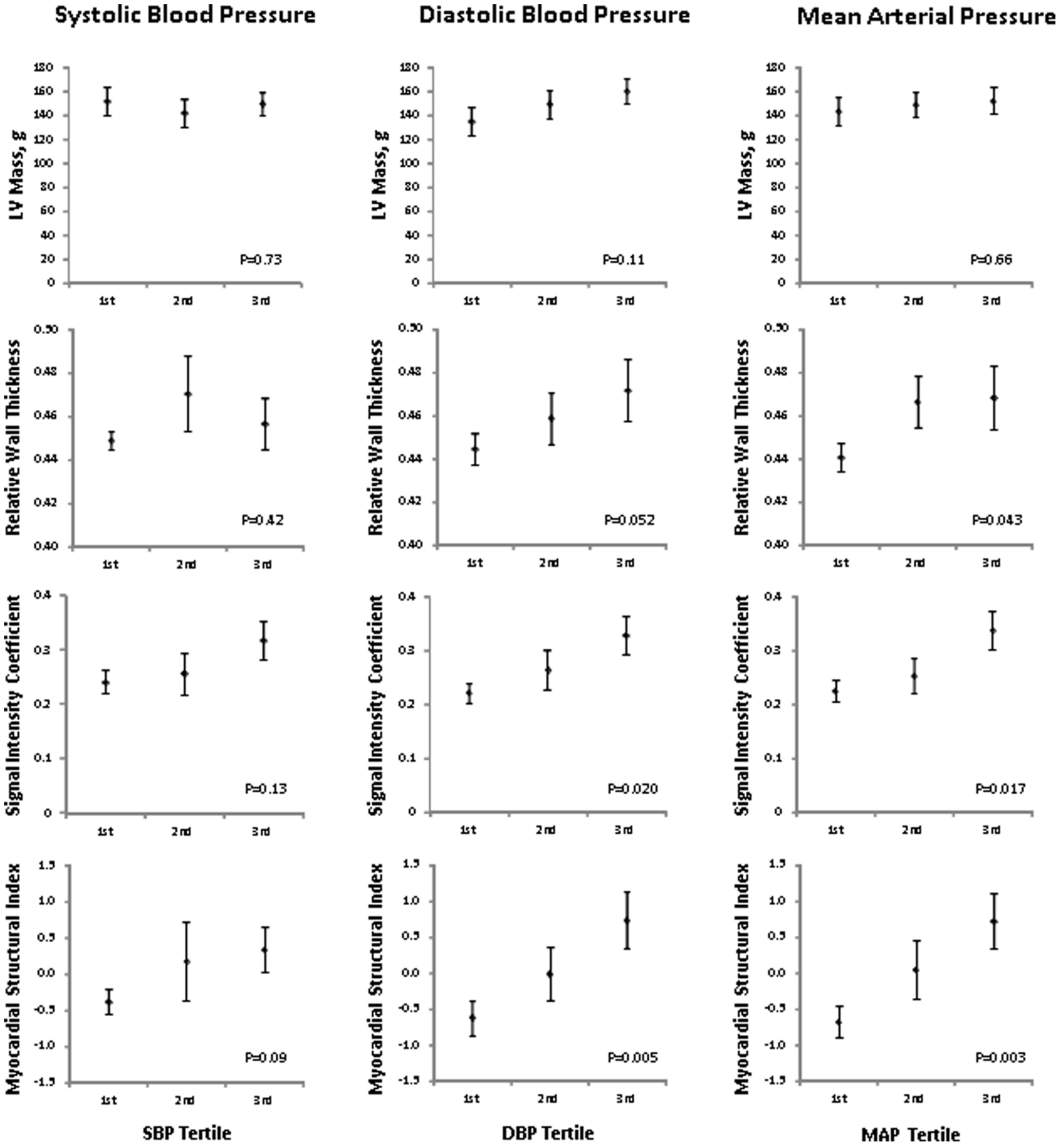
Mean (± standard error) values of conventional and advanced echocardiographic measures are displayed across increasing tertile of systolic, diastolic, and mean arterial pressure in the total study sample. Conventional echocardiographic measures include left ventricular mass and relative wall thickness; advanced measures include the signal intensity coefficient and myocardial structural index. P values are for non-parametric tests of trend cross tertiles of each blood pressure measure.

In analyses of variation in BP across all the individuals studied, there was no significant association of SBP, DBP, or MAP with LV mass (**Figure 1**). Although higher MAP was associated with higher RWT (P = 0.043), there was a borderline significant association of DBP with RWT (P = 0.052) and there was no significant association of SBP with RWT (P = 0.42). Notably, higher levels of SBP were also not significantly associated with either SIC or MSI in these unadjusted analyses. However, increasing DBP was associated with higher measures of both SIC (P = 0.020) and MSI (P = 0.005). Similarly, increasing levels of MAP was also associated with higher values of both SIC (P = 0.017) and MSI (P = 0.003).

In regression analyses examining the relation of BP indices with echocardiographic traits, there were no significant associations of BP with the following conventional measures of structure: LV size, total wall thickness, or mass (**Table 3**). The only associations observed between BP and any conventional measures included RWT with MAP (P = 0.02), and E prime with SBP (P = 0.03). By contrast, increasing values of SBP, DBP, and MAP were all significantly associated with measures of both the SIC and MSI (**Table 3**). These associations all remained significant in analyses adjusting for age and sex (P<0.05 for all), with the exception of a borderline significant relation of SBP with SIC (P = 0.053).

**Table 3:** Associations of Echocardiographic Parameters with Blood Pressure Indices.

	Systolic Blood Pressure		Diastolic Blood Pressure		Mean Arterial Pressure	
	Est. Coeff (SE)*	P value	Est. Coeff (SE)*	P value	Est. Coeff (SE)*	P value
***Unadjusted***						
LVWT	2.73 (2.57)	0.29	3.83 (1.67)	0.025	4.74 (2.26)	0.041
LVDD	−0.23 (2.62)	0.93	0.90 (1.76)	0.61	0.83 (2.37)	0.73
LV mass	1.12 (2.63)	0.67	2.61 (1.74)	0.14	2.98 (2.36)	0.21
RWT	3.61 (2.44)	0.14	3.92 (1.59)	0.017	5.12 (2.14)	0.020
E prime	−5.75 (2.58)	0.030	0.56 (1.82)	0.76	−1.36 (2.44)	0.58
E/a ratio	−1.88 (2.54)	0.46	0.92 (1.72)	0.59	0.30 (2.32)	0.90
E/e' ratio	4.40 (2.43)	0.08	−0.54 (1.68)	0.75	0.93 (2.26)	0.68
SIC	5.45 (2.40)	0.027	4.05 (1.60)	0.014	5.87 (2.14)	0.008
MSI	6.06 (2.36)	0.013	5.31 (1.52)	0.001	7.33 (2.04)	0.001
***Adjusted for age and sex***						
LVWT	2.30 (2.80)	0.41	2.34 (1.85)	0.21	3.11 (2.52)	0.22
LVDD	−1.48 (2.87)	0.61	−1.49 (1.90)	0.44	−1.98 (2.59)	0.45
LV mass	0.03 (2.95)	0.99	0.55 (1.96)	0.78	0.55 (2.67)	0.84
RWT	3.69 (2.36)	0.12	3.85 (1.51)	0.014	3.80 (1.59)	0.021
E prime	−6.05 (2.89)	0.041	−1.44 (2.00)	0.48	−2.98 (2.06)	0.16
E/a ratio	−1.75 (2.53)	0.49	0.11 (1.69)	0.95	−0.51 (1.77)	0.78
E/e' ratio	3.94 (2.60)	0.14	0.72 (1.77)	0.69	1.79 (1.84)	0.33
SIC	4.79 (2.41)	0.053	3.34 (1.60)	0.042	3.83 (1.66)	0.026
MSI	5.64 (2.33)	0.019	4.79 (1.49)	0.002	6.67 (2.02)	0.002

LVWT, left ventricular wall thickness; LVDD, left ventricular end-diastolic diameter; RWT, relative wall thickness; SIC, signal intensity coefficient; MSI, myocardial structural index.

*Estimated regression coefficients represent the change in blood pressure measure per 1-SD change in the echocardiographic parameter.

### Experimental Study Results

**Figure 2:**
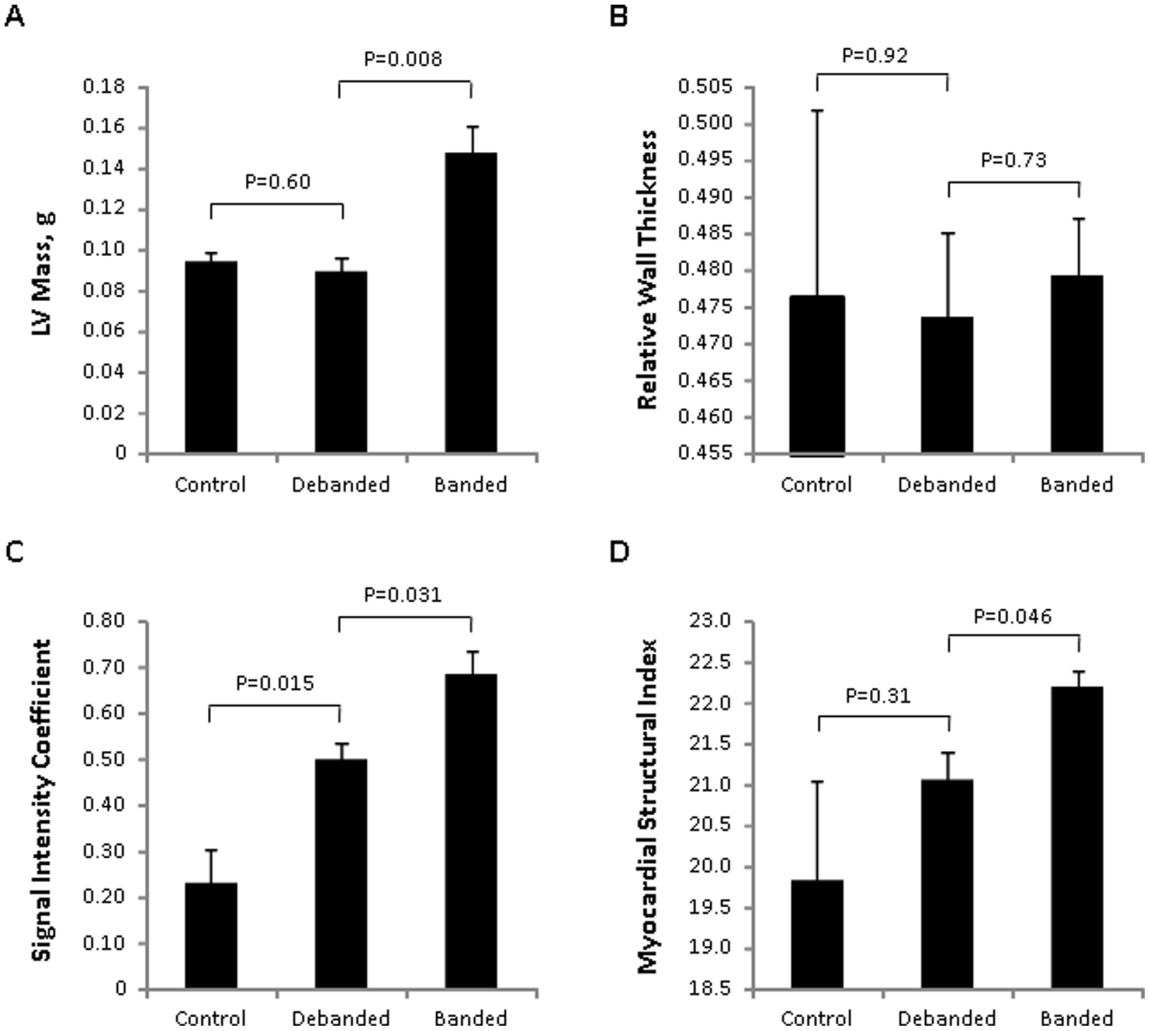
Mean (± standard error) values of conventional and advanced echocardiographic measures are displayed for each group of mice. : vehicle control, banded and then debanded mice, and continually banded mice. Conventional echocardiographic measures include left ventricular mass (Panel A) and relative wall thickness (Panel B). Advanced measures include the signal intensity coefficient (Panel C) and myocardial structural index (Panel D). P values are for Kruskal-Wallis tests of difference between independent groups.

**Figure 3:**
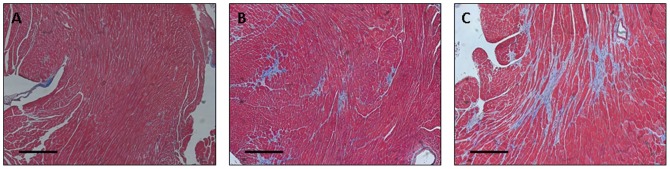
Histologic examination with Masson's trichome staining of myocardial tissue is shown for representative mice from each of the 3 animal groups. : control (Panel A), debanded (Panel B), and banded (Panel C). The black horizontal scale bars represent 50 micrometers.

After 7 weeks of the experimental protocol, echocardiographic images were acquired and analyzed for all 3 animal groups: mice that underwent the sham surgery (control), mice that underwent ascending aortic constriction at baseline and then debanding at 3 weeks (debanded), and mice that underwent ascending aortic constriction at baseline without further intervention (banded). With respect to conventional echocardiographic parameters (**Figure 2**), measures of LV mass differentiated between debanded and banded mice (P = 0.008), but not between control and debanded mice (P = 0.60) or across the 3 groups overall (P = 0.053). There was no significant difference between animal groups with respect to RWT (P≥0.73). By contrast, the SIC was significantly different across all 3 groups of mice (P = 0.026) and also differentiated between control and debanded (P = 0.015) as well as between banded and debanded mice (P = 0.031). The MSI differentiated between debanded and banded mice (P = 0.046) although not between control and debanded mice (P = 0.31); the MSI was not significantly different across all 3 groups of mice (P = 0.14). At the end of the protocol, mice from the control, debanded, and banded groups were sacrificed and histologic examination showed evidence of greater fibrosis in debanded compared with control mice, and greater fibrosis in banded compared to debanded mice (**Figure 3**). Image analysis-based automated quantification of myocardial fibrosis from representative Masson's trichrome stained sections demonstrated fibrosis scores of 3.2%, 8.1%, and 23.3% for control, debanded, and banded mice, respectively. All echocardiographic measures were highly correlated with quantitation of fibrosis: r = 0.95 for LV mass, r = 0.72 for RWT, r = 0.92 for SIC, and r = 0.95 for MSI.

## Discussion

We evaluated an ultrasound-based image analysis algorithm designed to differentiate microstructural characteristics of LV myocardium exposed to varying degrees of afterload stress. We also compared this algorithm with conventional echocardiographic parameters of adverse LV remodeling. The main findings of our study were three-fold. First, we observed that the SIC, an indirect measure of LV microstructure, was significantly higher in hypertensive compared to non-hypertensive myocardium in both our clinical study and experimental model. Second, the SIC was positively related to increasing levels of blood pressure exposure in humans as well as to increasing levels of afterload stress in mice. Third, we also observed in humans that the SIC demonstrated stronger associations with hypertension status and degree of blood pressure elevation than established echocardiographic measures of adverse LV remodeling (i.e. ‘macrostructure’). Overall, these results demonstrate the potential of an imaging algorithm to identify the presence and extent of microstructural changes that can arise early in the development of hypertensive heart disease.

In our clinical study, the individuals with hypertension were otherwise healthy and were similar to normotensive controls with respect to age and sex. Thus, the clinical cohort included younger to middle-aged individuals, in whom elevations in DBP are known to be more common than elevations in SBP.[20], [21] Accordingly, we observed that the SIC was more prominently associated with DBP and MAP rather than with SBP. Similar findings were seen for RWT, although these associations were not statistically significant. Thus, the SIC appears more sensitive to myocardial changes that are likely to occur during the earliest stages of variation in blood pressure. Consistent with our findings in humans, the SIC measurement performed in mice was able to differentiate between control and debanded, as well as between debanded and continuously banded, animals. In effect, the SIC was able to identify differences between animals exposed to mild versus moderate, as well as moderate versus severe, levels of chronic afterload resistance. Conversely, conventional echocardiographic measures of gross morphology (i.e. ‘macrostructure’), such as LV mass, only distinguished animals exposed to the highest levels of chronic afterload resistance. Taken together, our findings suggest that the SIC measure could serve as a sensitive marker of LV microstructure that complements current echocardiographic techniques for assessing the presence and severity of changes that arise along the spectrum of hypertensive heart disease.

The image analysis algorithm used to produce the SIC measure differs in several ways from similar algorithms employed in prior studies. First, the SIC is based on the 25^th^ percentile of the signal distribution in the region of interest. Many published techniques for assessing myocardial microstructure have relied on mean gray-scale values, obtained through integrated backscatter or comparable signal analysis methods. However, other studies have suggested that using the complete distribution of signal intensity values allows for a more sensitive and specific assessment of signal alterations compared with use of only the mean signal intensity value. Ciulla et al. demonstrated that kurtosis and other measures of signal distribution were positively correlated with collagen content in hypertensive patients.[22] In a similar fashion, a percentile value of signal intensity offers information regarding the widening or shift of a signal distribution, in addition to allowing for quantitative comparisons between measures. Second, the SIC measure represents signal intensities within a pericardial region of interest. Prior studies have traditionally assessed the myocardium, alone or standardized to the pericardium. Cyclical variation of the myocardial signal (i.e. standardization of a myocardial measures in one frame compared with another) has also been assessed. Use of a pericardial region of interest extends from these prior methods given the fact that ultrasound signal reaching and reflected by the pericardium is dependent upon its transmission through the adjacent myocardium. Thus, in the standard orientation of sonographic transduction in the long axis view, the pericardial signal is a function of the post-myocardial signal.

The exact mechanism by which the SIC measure is able to differentiate between states of afterload resistance is not entirely clear. Studies of microstructural image analysis have long sought to understand how pathologic changes of the myocardium manifest as sonographic signal intensity variation. Prior investigators using echocardiographic analyses of hypertensive disease have suggested that pathologic compared with non-pathologic changes within the LV wall can effectively change tissue impedances, thereby altering sonographic signals. Several studies have noted that cyclic variation in mean gray levels of integrated backscatter signals, which normally increase at end-diastole and decrease at end-systole, show less variation in the setting of hypertension and coronary disease.[3], [5], [6], [23] Tissue-level characteristics that influence ultrasonic backscatter are thought to include collagen content and fibrosis, tissue heterogeneity, fiber orientation, wall thickness, cell size, and sarcomere length.[3]–[5], [22] Interestingly, histologic changes seen in hypertension have been associated with both increases and decreases in signal density. Increased signal intensity has been attributed specifically to wall thickness and increase myocyte size and density.[24] Leftward shifts (i.e. decrease) in signal intensity have been attributed to heterogeneously increased collagen content and fiber disorientation that can cause constructive and destructive signal interactions with diffuse signal scattering[22].

In the present study, the positive correlation observed between SIC and BP indicates that a pre-specified percentile value of pericardial signal intensity decreases as BP increases. Signal that is more diffusely scattered due to increased tissue heterogeneity and fiber disarray may demonstrate lower signal intensity upon reaching the interface of the myocardium and pericardium. Alternatively, signal that is reflected in the myocardium more proximally, due to collagen or calcium deposits, may also result in reduced pericardial intensities. This kind of “acoustic speckle” effect has been described previously[25] and may be similar to the acoustic shadowing effects seen in ultrasound image analysis studies of vascular atherosclerosis.[26], [27] Additionally, it is possible that some microstructural features influence the signal intensity more than others. Distinct microstructural changes may appear at different stages during the progression of hypertensive heart disease, potentially contributing to the inconsistent findings reported by prior studies of myocardial signal intensity in the setting of hypertension. Thus, analysis of the myocardium may be affected by both scattering effects that reduce the signal and increased echogenicity of structures that increase the signal. By contrast, the pericardial signal is likely to reflect only the changes due to scattering, yielding a uniformly lower signal. Further studies are needed to elucidate the exact mechanisms by which the pericardial value appears to consistently decrease and, in turn, the SIC is observed to consistently increase in relation to higher levels of afterload resistance.

### Comparison with Conventional Echocardiographic Measures

The present study extends prior work by comparing and integrating established measures of structural heart disease with ultrasonic measures of LV myocardial microstructure. Although conventional echocardiographic measures, such as LV mass and wall thickness, are known to reflect worsening hypertensive heart disease, the relations of these gross morphologic parameters with BP exposure are non-linear and complex.[1] Because microstructural changes are likely to precede macrostructural changes of the myocardium, a microstructural marker has the potential to identify alterations in remodeling both earlier and with greater sensitivity in the setting of BP exposure. Furthermore, when a microstructural marker such as the SIC is combined with a macrostructural measure such as RWT, an aggregate measure such as the MSI offers the potential to characterize myocardial changes across the wider spectrum of varying levels of BP exposure. Accordingly, we observed particularly significant associations of BP status with the MSI in humans. The MSI was less discriminating in mice, particularly in comparisons of animals exposed to mild versus no afterload resistance; this finding is likely due to fact that the MSI is dependent on wall thickness measures which are less precise in mice than in humans

### Potential Applications

The SIC and the MSI measures offer the potential to serve as markers of myocardial disease severity in the assessment of hypertensive heart disease in humans, including changes in myocardial structure over time and responses to treatment. Advantages of the SIC and MSI include their wide accessibility and ease of use through open access software that can be applied to routinely acquired echocardiographic images. Although further evaluation in more diverse clinical cohorts is warranted, our initial results in a human sample and in an animal model suggest that the SIC may be particularly robust to variation in image acquisition settings and physiology. As a measure of cardiac microstructure, the SIC may also be used to differentiate the severity of myocardial pathology in non-hypertensive disease states. Further studies are needed to assess the extent to which the SIC correlates with histologic changes in myocardial microstructure during the course of disease progression. Further studies are also needed to assess the potential utility of the SIC as well as MSI in broader clinical contexts. In addition to potential clinical applications, validated microstructural image analysis techniques may be used as adjunctive tools for investigating the pathophysiology of hypertensive heart disease. Hypertensive heart disease is recognized as a complex entity with multiple subclinical and clinical disease manifestations.[1] Thus, microstructural imaging techniques could be used to further study the time course and nature of myocardial changes at the tissue level in more diverse clinical cohorts.

### Limitations

Several limitations of the study merit consideration. Our study design was retrospective, although all analyses were performed while blinded to clinical characteristics. Additionally, our human study sample size was small, although findings in this small-sized cohort demonstrated significant associations between the novel ultrasonic measures of LV microstructure and hypertension status as well as BP variation; nonetheless, further studies in larger samples are warranted. Because BP is known to be difficult to measure in mice,[28] banded versus debanded status was used as a surrogate measure of variation in chronic afterload resistance in the experimental model. With respect to findings from the clinical study, generalizability to populations of varying age group, race, and comorbidities remains unknown and may be the subject of future investigations.

### Conclusion

We evaluated a novel image analysis algorithm designed to assess alterations in cardiac microstructure due to hypertension. We observed that the SIC significantly differentiates between hypertensive and non-hypertensive myocardium, both in humans and in mice. Furthermore, the SIC measure correlated with even mildly increased levels of BP exposure in humans, as well as with increasing levels of chronic afterload stress in mice. Overall, the SIC demonstrated stronger associations with the presence and degree of hypertension exposure when compared to established echocardiographic measures. Thus, the SIC may represent a particularly sensitive measure of cardiac remodeling that adds complementary information to conventional methods for assessing myocardial changes occurring during the progression of hypertensive heart disease.

## Supporting Information

File S1 **File S1 contains additional information relevant to the manuscript, including the Protocol for Image Analysis, Figure S1 (selection of the pericardial region of interest), and Figure S2 (example of signal intensity coefficient measures performed for images acquired at different gain settings).**
(DOC)
