# Supplementary material for: Identifying Early Changes in Myocardial Microstructure in Hypertensive Heart Disease
Source: PLoS One. 2014 May 15;9(5):e97424. doi: 10.1371/journal.pone.0097424 (PMC4022613; doi:10.1371/journal.pone.0097424)
Supplement: File S1 — contains additional information relevant to the manuscript, including the Protocol for Image Analysis, Figure S1 (selection of the pericardial region of interest), and Figure S2 (example of signal intensity coefficient measures performed for images acquired at different gain settings). (DOC) [file pone.0097424.s001.doc]

**SUPPORTING INFORMATION**

**Protocol for Image Analysis**

1. ***Prepare images***
   1. Use human or mouse echocardiographic images in the B-mode parasternal long-axis view. Per usual practice, optimize visualization of cardiac structures in the parasternal view by adjusting placement of the transmit focus and the time-gain compensation settings. Images must be saved in DICOM file format. Standardized images place the inferolateral left ventricular wall near the base of the frame. Frames should display the complete LV myocardium and pericardium. Ensure that resolution is high enough to demarcate the LV endocardial border, myocardial wall, and pericardial border. Discard any images with artifacts or excess dropout.
   2. Use an echocardiographic viewing program to isolate the end-diastolic frame of your image file. Export to a high-resolution .jpg file, and then import into ImageJ software platform v1.46 (<http://rsb.info.nih.gov/ij/>). Convert to an 8-bit file.
   3. Consider ensuring that the readers who select the regions of interest are blinded to clinical and hemodynamic information about the study subject.
2. ***Sample the region of interest***
   1. Pericardial region of interest (ROI) selection: aim to capture the heterogeneity of the bright pericardial tissue. Note that contrast and brightness may be adjusted without influencing results of the analysis.
   2. Select a rectangle approximating the middle third of the mid-to-basal inferolateral pericardial wall, using ImageJ’s rectangle drawing tool.
   3. Resize the ROI to span the width of the pericardium, using ImageJ’s ROI resizing tool. Rotate the ROI as necessary to lie within the pericardial region, parallel to what would be an adjacent mid-myocardial selection of the myocardium at the same level (**Supplemental Figure S1**).
   4. The final ROI should lie within the middle third of the pericardium and should include the width of the wall without extending into surrounding regions. All measures made in a given study should have similar relative location, theta angle, and percentage of total pericardial area. Relative differences in gain of the selected image should not substantially affect the pericardial measurement value for a given patient (**Supplemental Figure S2**).
3. ***Analyze data***
   1. Apply the algorithm via an ImageJ macro.
   2. Arrange the intensity values hierarchically. Determine the 25^th^ percentile of these intensity values.
   3. Calculate the Signal Intensity Coefficient (SIC) by using the formula 1-p/256, where p is the 25^th^ percentile of the signal intensity values.

**SUPPLEMENTAL FIGURES LEGEND**

**Supplemental Figure S1.** Selection of the pericardial region of interest is show in red, adjacent and parallel to a mid-myocardial selection of the mid-to-basal inferolateral segment of left ventricle in the parasternal long axis view.

**Supplemental Figure S2.** Repeated measures of the signal intensity coefficient (SIC) for representative patients do not substantially vary based on within-patient differences in gain.

**Supplemental Figure S1.**

**Supplemental Figure S2.**
